# Good reasons for bad behavior: a randomized controlled experiment on the impact of narrative reading and writing on empathic concern, perspective-taking, and attitude

**DOI:** 10.3389/fpubh.2024.1343225

**Published:** 2024-04-05

**Authors:** Martina Bientzle, Marie Eggeling, Joachim Kimmerle

**Affiliations:** ^1^Knowledge Construction Lab, Leibniz-Institut für Wissensmedien, Tuebingen, Germany; ^2^Department of Psychology, Eberhard Karls University Tuebingen, Tuebingen, Germany

**Keywords:** narrative writing, narrative reading, perspective-taking, empathic concern, attitude, stigmatization

## Abstract

**Background:**

Empathic concern and perspective-taking may contribute to avoiding stigmatization of adverse health behavior. Narrative writing has been shown to be effective in promoting perspective-taking and empathy. But since narrative writing is time consuming, we tested in the present study narrative reading as an alternative, more parsimonious approach.

**Methods:**

In a randomized controlled experiment, we compared writing a narrative text about a fictitious person who displays disapproved of health behavior to reading such a text and to a control condition in which participants wrote about an unrelated topic. With a sample of *n* = 194 participants, we investigated the impact of writing and reading a narrative text on promoting empathic concern and perspective-taking as well as on attitude change.

**Results:**

We found that both writing and reading a narrative text about the fictitious character increased empathic concern, *F*_(1, 191)_ = 32.85, *p* < 0.001, part. η^2^ = 0.15, and perspective-taking, *F*_(1, 191)_ = 24.76, *p* < 0.001, part. η^2^ = 0.12, more strongly than writing about an unrelated topic. Writing and reading a narrative text also resulted in a more positive attitude toward this person, *F*_(1, 191)_ = 17.63, *p* < 0.001, part. η^2^ = 0.08. Simply reading a narrative text was equally efficient as narrative writing with respect to empathic concern, *p* = 0.581, perspective-taking, *p* = 0.629, and attitude, *p* = 0.197.

**Conclusion:**

The finding that narrative reading is as effective as narrative writing suggests that the readers appear to be able to comprehend and engage with the story being told. When narrative reading is as effective as narrative writing, it can succeed with reduced effort in increasing empathic concern, perspective-taking, and attitude. We discuss the benefits of this approach for reducing stigmatization of adverse health behavior.

## 1 Introduction

Many health campaigns aim to reduce negative health behavior in people like drinking too much alcohol, eating sugary food, driving too fast, or smoking while being pregnant (1–3). Nevertheless, a lot of people exhibit negative health behavior at some point in their lives. Observing this behavior can be disturbing to observers and lead to negative attitudes toward the person who is exhibiting this behavior. It is well known that negative attitudes toward other people have far-reaching consequences in many life situations, like the interaction with peers, colleagues, or family members (4); it has been shown that the attitude of employers can be a barrier to employment (5). Since negative health behavior often results in medical treatment, the impact of negative attitude of health care providers toward patients is especially important (6–9). “There is great concern that the attitudes and beliefs of providers will influence the practice of health care and contribute to the documented health disparities” [(10); p. 2]. Therefore, interventions are required facilitating that people are treated without stigmatization in the immediate personal and wider social environment as well as in the health care system.

Empathic concern and perspective-taking may be important for avoiding stigmatization. Empathic concern is an other-oriented emotion that includes a positive worry over another person, whereas perspective-taking comprises the adoption of other people's point of view and seeing things from their perspectives (11). Empathic concern and perspective-taking could mitigate stigmatization because they foster understanding, combat stereotypes, promote social inclusion, reduce discrimination, and build empathic communities (12–14) [even though empathy has also a downside (15) and has been connected to selfishness (16) and the manipulation and deception of others (17)]. By empathizing with individuals who face stigmatization, people can challenge prejudice, advocate for equality, and create environments where everyone is treated with dignity and respect. There is evidence that narrative writing may be an effective intervention to promote perspective-taking and empathic concern as two basic capabilities for a good communication basis and for fair behavior (10, 18). Even in the context of strong political opinions, narrative writing strategies have the power to improve attitudes toward the outgroup and reduce political polarization (19). Based on the cognitive processing theory of writing (20), the writing process helps writers to make sense, organize their thoughts, and come to sensible conclusions. However, narrative writing is very time consuming and demanding, and thus not suitable for the busy and stressful daily schedule in the health care system. One potentially promising approach more suitable for everyday life is the idea of simply reading fictional narratives. Reading narratives may help people to develop empathic concern for the persons described in the story who show stigmatized behavior. Also, when reading, the readers may imagine the circumstances and specific stressors of people who show negative health behavior (10, 21, 22).

There is preliminary evidence that reading narratives can have an impact on perspective-taking, empathy, and attitude. A previous study has shown that medical experts who read a patient narrative were more willing to engage in shared decision making and to plan more time for medical conversation than medical experts who have read only factual information about the patient (23). Narrative writing and narrative reading are assumed to promote perspective-taking and empathic concern through several mechanisms (10, 12, 23). These mechanisms include facilitating identification with the characters depicted in a narrative text and eliciting emotional engagement as people may vicariously experience the joys, sorrows, and hardships encountered by these characters. Perspective-shifting may also be encouraged, enabling individuals to adopt alternative viewpoints and consider the world from different perspectives (12). Through these mechanisms, individuals may develop a deeper understanding of others' perspectives and experiences, enhancing their capacity for empathic concern and compassionate engagement. In the present study, we aimed to directly compare reading narrative texts as a less time consuming and demanding strategy with the well-studied strategy of narrative writing.

We conducted an experimental study to examine whether writing a fictional narrative text about a person who engages in a stigmatized health-related behavior (smoking while pregnant) vs. reading such a narrative text vs. writing about something different (control group) affects people's empathic concern, perspective-taking, attitude toward this person, and attribution of the stigmatized behavior. Given the theoretical background described above, we stated and pre-registered the following hypotheses:

*Hypothesis 1*. We hypothesized that writing or reading a fictional narrative text would increase empathic concern (H1a) and perspective-taking (H1b), improve the attitude toward this person (H1c), and make participants attribute that behavior more strongly to the role of external factors of influence (H1d) compared to the control condition.

*Hypothesis 2*. Because writing requires a more active and mindful engagement with the subject matters than reading, we also hypothesized that writing a narrative text would show stronger effects than reading a narrative text, again regarding empathic concern (H2a), perspective-taking (H2b), attitude (H2c), and attribution (H2d).

## 2 Materials and methods

The experiment that is presented here was part of a larger research project that was approved by the Ethics Committee of the Leibniz-Institut für Wissensmedien (approval number: LEK 2020/032). The study presented in this article was preregistered on the preregistration platform AsPredicted (aspredicted.org) before we began data collection; registration number: #44755; https://aspredicted.org/fq3ek.pdf.

### 2.1 Sample

A power analysis for ANOVAs with α = 0.05, an intended power of 95%, and an assumed medium effect size of f = 0.25 indicated a required sample size of 189 participants (for main effects of condition). We also indicated that we would exclude those participants (1) who implied that they were not sufficiently motivated during their participation in the study, (2) who did not have suitable German language skills, or (3) who suggested that the recommendation not to smoke during pregnancy would not make sense to them (i.e., did not recognize smoking during pregnancy as adverse health behavior). We did not at all invite people who were younger than 18 years old or who did not speak German fluently.

**Figure 1:**
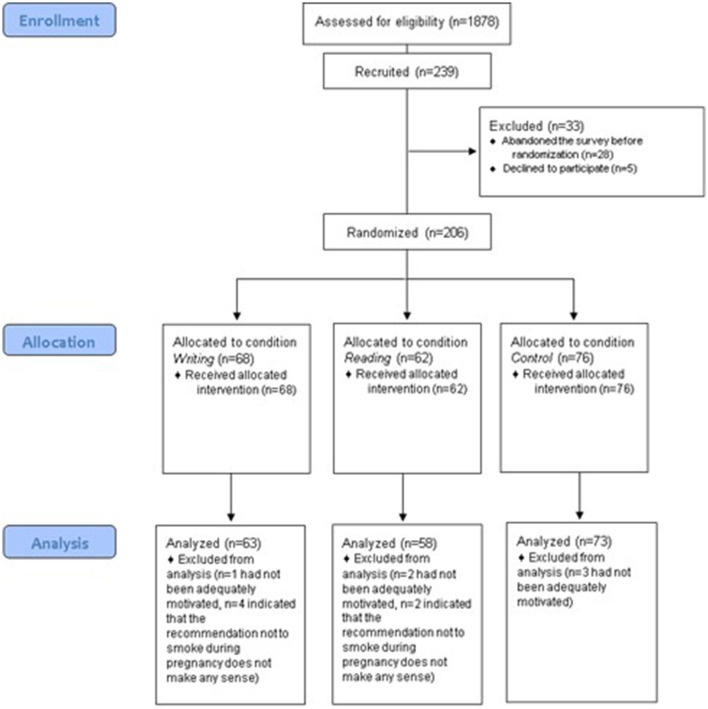
Sampling procedure.

We used the online participant recruitment platform Prolific (https://www.prolific.com/) to recruit the participants for this study. There were 1,878 potential participants who fulfilled the inclusion criteria and were thus invited to participate in our study. Two hundred and nine people started participation in the study; 45 participants had to be excluded from the data analysis as they did not provide their written informed consent (*n* = 5), canceled the survey before being allocated to the manipulation procedure (*n* = 28), indicated that they had not been sufficiently motivated to participate (*n* = 6), or that the recommendation not to smoke during pregnancy would not make sense (*n* = 6). After these exclusions, the data from the remaining *N* = 194 participants were analyzed (writing condition: *n* = 63 participants; reading condition: *n* = 58; control condition: *n* = 73 participants). A detailed overview of the sampling procedure can be seen in Figure 1.

Across the entire sample, there were 84 females, 109 males, and one non-binary person. On average, participants were *M* = 29.69 years old (*SD* = 10.07; age range: 18–68 years). There were no group differences regarding gender (χ^2^ = 2.303, *p* = 0.680) or age [*F*_(2, 191)_ = 0.228, *p* = 0.796] among the three experimental conditions. Since it is conceivable that smokers would judge the health-related behavior (smoking while pregnant) differently than non-smokers, we asked the participants as control variables whether they were smokers themselves and whether their mothers were smokers at any point during the participants' childhood. There were no group differences regarding these control variables (smoker themselves: χ^2^ = 1.791, *p* = 0.408; mother smoker: χ^2^ = 2.433, *p* = 0.657) among the three conditions. A detailed overview of demographics and smoking status in the study sample is shown in Table 1.

**Table 1:** Demographics and smoking status in the study sample.

**Measures**	**Writing condition**	**Reading condition**	**Control condition**
	**(*****n** =* **63)**	**(*****n** =* **58)**	**(*****n** =* **73)**
Gender	Male: *n =* 37	Male: *n =* 34	Male: *n =* 38
	Female: *n =* 26	Female: *n =* 24	Female: *n =* 34
	Non-binary: *n =* 0	Non-binary: *n =* 0	Non-binary: *n =* 1
Age	*M =* 29.27, SD = 9.14	*M =* 30.43, SD = 10.72	*M =* 29.47, SD = 10.41
	Range: 18–57	Range: 18–68	Range: 18–63
Smoking status	Smoker: *n =* 5	Smoker: *n =* 9	Smoker: *n =* 10
	Non-smoker: *n =* 58	Non-smoker: *n =* 49	Non-smoker: *n =* 63
Mothers' smoking status	Smoker: *n =* 21	Smoker: *n =* 15	Smoker: *n =* 23
	Non-smoker: *n =* 40	Non-smoker: *n =* 42	Non-smoker: *n =* 46
	Not known: *n =* 2	Not known: *n =* 1	Not known: *n =* 4

### 2.2 Procedure

The study procedure, instructions, and measures were adopted from studies by Shaffer et al. (10) as well as Bientzle et al. (18). As an extension to these previous studies, the study design presented here was refined by the addition of a reading condition. To conduct the study online, we used the participant recruitment platform Prolific and the online tool Qualtrics Survey Software (Provo, Utah, USA). The randomization procedure was conducted by the Qualtrics Survey Software's random generator with no humans being involved in the allocation. Before starting the survey, participants provided their written informed consent. Then we presented the fictional scenario of seeing a pregnant woman who is smoking a cigarette in a parking lot in front of a supermarket. Then participants answered questions regarding empathic concern, perspective-taking, attitude, and attribution of causes regarding this fictitious person's behavior. After that, the participants were randomly assigned to one of the three conditions.

In the *writing condition*, they received the instructions to compose a narrative text about the pregnant woman. The participants were instructed to imagine this person in a concrete way and to think about possible information about her (such as her name, age, living situation, etc.). The only constraint was that the pregnant woman should be considered to be at least as intelligent as the participants themselves. Then we asked participants to dedicate at least 10 min to write down their text. An automatic timer in the survey prevented them from continuing to the next page sooner than after 5 min. Participants spent *M* = 23.80 min (SD = 8.79, range: 9.25–48.15 min) on the task in this condition. Participants' texts had a length of *M* = 217.45 words (SD = 75.77, range: 87–348 words).

In the *reading condition*, they received the following instruction: “Earlier we asked you to imagine that you are leaving a grocery store and see a pregnant woman smoking a cigarette in the parking lot. Please think about this woman. Next, you will receive a fictional text written by another person. The scene is about the woman you saw in the parking lot. Please read the text and try to put yourself in the position of the main character of the text.” Then participants read a narrative text about the woman, which was presented on a computer screen (an English translation of this text can be found in Supplementary material 1). Participants spent *M* = 12.29 min (SD = 4.73, range: 6.87–32.65 min) on the task in this condition.

In the *control condition*, participants received the instructions to write a text about the room they were staying in during the study. They also were requested to dedicate at least 10 min to write this text. An automatic timer prevented them from continuing before 5 min of the writing time had passed. Participants spent *M* = 19.47 min (*SD* = 15.15, range: 9.26–126.63 min) on the task in this condition. Participants' texts had a length of *M* = 182.63 words (*SD* = 68.98, range: 82–326 words).

After completing the respective tasks, participants in all of the three conditions answered again the same questions regarding empathic concern, perspective-taking, and attitude. Unfortunately, we made a mistake when creating the study material, with the result that the items regarding attribution were not identical in the pre- and the posttests. Due to this lack of comparability, we had to exclude the attribution items from further analysis. In addition, participants were asked if the recommendation not to smoke during pregnancy would make sense to them, about their motivation while taking part in the study, their demographic data (age and gender), as well as the smoker status of themselves and their mother.

### 2.3 Material

We used the fictional scenario developed by Shaffer et al. (10). This scenario described the situation of seeing a pregnant woman smoking a cigarette in a parking lot in front of a supermarket. The current study aimed at replicating and extending the findings by Shaffer and colleagues by using an additional study condition (i.e., the reading condition).

### 2.4 Measures

All dependent variables were assessed twice; initially after participants read the description of the situation (t1) and again after they wrote (writing condition; control condition) or read (reading condition) a text (t2).

Empathic concern and perspective-taking were measured by a German language adaptation (24) of the Interpersonal Reactivity Index (25). It consists of four subscales: perspective-taking, empathic concern, fantasy, and personal distress. In line with the study by Shaffer et al. (10), we used the two subscales perspective-taking and empathic concern in the present study. The four perspective-taking items measured people's capability to put themselves in someone else's place in terms of a cognitive ability. The four items on empathic concern captured people's involvement in someone else's feelings in terms of an emotional capability (11). The items were adapted to the fictional situation of the pregnant woman (the original items (25) can be seen in Table 2).

**Table 2:** Measurement of perspective-taking and empathic concern.

**Perspective-taking**		**Empathic concern**	
Wording used in the present experiment	Original item wording (25)	Wording used in the present experiment	Original item wording (25)
1). I tried to imagine her perspective of the problem.	I sometimes try to understand my friends better by imagining how things look from their perspective.	1). I felt the need to protect the woman.	When I see someone being taken advantage of, I feel kind of protective toward them.
2). I tried to look at the woman's perspective in addition to my own.	I believe that there are two sides to every question and try to look at them both.	2). Just imagining the situation has emotionally touched me.^*^	I am often quite touched by things that I see happen.
3). Before I criticized the woman, I tried to imagine how I would feel if I were in her place.	Before criticizing somebody, I try to imagine how I would feel if I were in their place.	3). I would consider myself a pretty soft-hearted person, based on how I felt when I imagined the scene.	I would describe myself as a pretty soft-hearted person.
4). When the woman's behavior seemed strange to me, I tried to put myself in her shoes.	When I'm upset at someone, I usually try to “put myself in his shoes” for a while.	4). I had tender feelings for the woman.	I often have tender, concerned feelings for people less fortunate than me.

^*^not used for analysis because of low internal consistency.

Participants indicated their agreement to the items on a scale ranging from “I don't agree at all” (0) to “I fully agree” (100). For the perspective-taking sub-scale, the internal consistencies were excellent (Cronbach alpha α = 0.90 at t1 and α = 0.91 at t2). Internal consistencies for the empathic concern sub-scale at t1 was insufficient (Cronbach alpha α = 0.55 at t1); without item 2 the internal consistencies were acceptable to good, however (Cronbach alpha at t1: α = 0.65 and t2: α = 0.75). Consequently, for the following analysis, the empathic concern score was calculated only from items 1, 3, and 4. All items are shown in Table 2.

**Figure 2:**
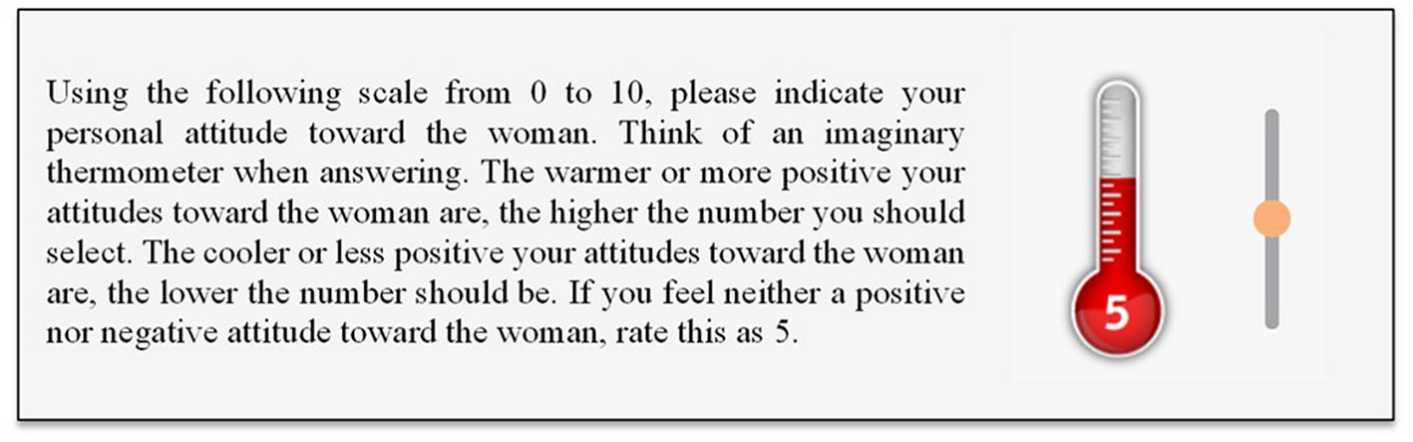
Feeling thermometer for capturing participants' attitude.

We used a feeling thermometer to capture participants' attitude (26). With this measure people rank their attitude toward the fictitious character on a scale ranging from 0 (negative attitude/“cold”) to 10 (positive attitude/“warm”), with a score of 5 indicating a neutral attitude (see Figure 2).

We conducted the data analysis employing IBM SPSS 25 statistics for Windows. Most variables were not normally distributed. Since simulation studies have disclosed that ANOVAs are robust to normal distribution violations (27, 28), we completed analyses of variance (ANOVA)—with repeated measure analysis, pairwise comparisons, and contrast analyses—to test our hypotheses. In the following results section, we present means (*M*) and standard deviations (*SD*); *F*-values, *p*-values, and partial eta-squared (part. η^2^) as an effect size metric.

## 3 Results

### 3.1 Empathic concern and perspective-taking

We found a significant increase in empathic concern for the participants across the conditions; *F*_(1, 191)_ = 90.67, *p* < 0.001, part. η^2^ = 0.32). At t1, participants indicated less empathic concern for the fictitious pregnant woman (*M* = 27.48, *SD* = 19.71) than at t2 (*M* = 37.92, *SD* = 22.49). There was also a significant interaction effect between time of measurement and condition, *F*_(2, 191)_ = 16.67, *p* < 0.001, part. η^2^ = 0.15). In Hypothesis 1a, we had assumed that writing or reading a narrative text about the pregnant woman would increase empathic concern more strongly than writing about an unrelated topic. The result of a contrast analysis for this interaction effect with the contrast coefficients [0.5 0.5 −1] supported this hypothesis (H1a), *F*_(1, 191)_ = 32.85, *p* < 0.001, part. η^2^ = 0.15. The means and standard deviations for all dependent measures split by condition and time can be seen in Table 3.

**Table 3:** Means (*M*) and standard deviations (*SD*) for the dependent measures split by condition and time.

			***M***	***SD***
Empathic concern	Writing condition	t1	25.93	20.42
		t2	42.28	23.24
	Reading condition	t1	27.53	18.92
		t2	42.26	20.68
	Control condition	t1	28.79	19.86
		t2	30.71	21.63
Perspective-taking	Writing condition	t1	39.76	25.39
		t2	63.03	18.62
	Reading condition	t1	42.26	29.15
		t2	63.61	22.23
	Control condition	t1	40.89	28.91
		t2	47.04	29.94
Attitudes toward the fictitious person	Writing condition	t1	1.35	1.44
		t2	3.05	1.83
	Reading condition	t1	1.47	1.79
		t2	2.78	2.59
	Control condition	t1	1.51	1.70
		t2	1.99	1.83

We also found an increase in participants' perspective-taking across the conditions; *F*_(1, 191)_ = 114.77, *p* < 0.001, part. η^2^ = 0.38. At t1, they scored lower on perspective-taking (*M* = 40.93, *SD* = 27.76) than at t2 (*M* = 57.19, *SD* = 25.58). Again, there was a significant interaction effect between time of measurement and condition, *F*_(2, 191)_ = 12.56, *p* < 0.001, part. η^2^ = 0.12). In Hypothesis 1b, we had stated that writing or reading a narrative text about a fictitious pregnant woman would increase perspective-taking more strongly than writing about an unrelated topic. The result of a contrast analysis for this interaction effect with the contrast coefficients [0.5 0.5 −1] supported this hypothesis (H1b), *F*_(1, 191)_ = 24.76, *p* < 0.001, part. η^2^ = 0.12.

### 3.2 Attitude

There was a significant effect of time; *F*_(1, 191)_ = 95.86, *p* < 0.001, part. η^2^ = 0.33. At t1, the attitude was more negative (*M* = 1.44, *SD* = 1.64) than at t2 (*M* = 2.57, *SD* = 2.13) across the conditions. In addition, there was a significant interaction effect between time of measurement and condition, *F*_(2, 191)_ = 9.80, *p* < 0.001, part. η^2^ = 0.09. We had stated in Hypothesis 1c that writing or reading a narrative text about a pregnant woman would result in a more positive attitude toward this person than writing about an unrelated topic. Again, the result of a contrast analysis for this interaction effect with the contrast coefficients [0.5 0.5 −1] supported this hypothesis (H1c), *F*_(1, 191)_ = 17.63, *p* < 0.001, part. η^2^ = 0.08.

### 3.3 Impact of writing vs. reading

We hypothesized that writing a narrative text would show stronger effects than reading a narrative text. The result of a contrast analysis with the contrast coefficients [1 −1 0], however, did not supported this assumption regarding empathic concern (H2a), *F*_(1, 191)_ = 0.31, *p* = 0.581, perspective-taking (H2b), *F*_(1, 191)_ = 0.23, *p* = 0.629, or attitude (H2c), *F*_(1, 191)_ = 1.68, *p* = 0.197. Writing a narrative text did not have any stronger effects on empathic concern, perspective-taking, or attitude than simply reading a narrative text.

## 4 Discussion

The experiment presented here studied the impact of narrative writing and narrative reading interventions on people's empathic concern, perspective-taking, and attitude. In line with our preregistered hypotheses, empathic concern, perspective-taking, and attitude toward the fictitious character were modified more strongly in the narrative reading and writing groups than in the control condition. The impact of the reading and writing intervention on perspective-taking and attitude had a medium effect size, the impact on empathic concern even had a large effect. Contrary to our hypotheses, we found no statistically significant differences between the narrative reading and writing interventions. The finding that narrative writing supports empathic concern, perspective-taking, and attitude is in line with previous research (10, 12, 18); the finding that narrative reading is equally supportive extends previous insights in an important way. As the study had a good statistical power, we cautiously interpret this result as an actual non-existent difference between reading and writing a narrative text. Of course, it would be important to confirm this non-existing difference in future research. Until then, it can only be considered as a preliminary finding.

The finding that narrative reading is as effective as narrative writing suggests that the readers appear to be able to comprehend and engage with the story being told, in the same way as if they were the ones creating it themselves. This can be considered as beneficial since narrative reading allows readers to step into the shoes of the characters described and experience their emotions and perspectives. Previous research has already shown that it can be helpful to deal with the personal experiences and emotions of others in the health context (29, 30). This also applies to doctor-patient communication (31). Since the willingness to engage emotionally with other people can lead to increased empathic concern and understanding of others, it may reduce the risk of stigmatizing adverse health behavior. When readers are fully engaged with a story, they are also more likely to analyze and evaluate it critically. This can lead to enhanced critical thinking skills and the ability to identify themes and motifs. Overall, when narrative reading is as effective as narrative writing, it can succeed with reduced effort in increasing empathic concern, perspective-taking, and attitude.

This approach is particularly beneficial when it avoids stigmatizing adverse health behavior since such stigmatization can have several negative consequences (5, 6). Stigmatization can discourage people from seeking help. When certain behaviors are being stigmatized, it can create a sense of shame and guilt in people who engage in them (32). This can make it less likely that they will seek help or treatment for their health issues, for fear of being judged or stigmatized. Stigmatizing certain behaviors can also reinforce negative stereotypes and contribute to discrimination against certain groups of people (33). Moreover, stigmatization can create barriers to effective care. The stigmatization of certain behaviors can make it more difficult for people to access the care they need. This can happen when health professionals are reluctant to provide care to people who engage in stigmatized behaviors, or when policies and systems are designed in ways that make it difficult for these individuals to access care. Finally, stigmatization can worsen health outcomes by creating stress and other negative emotions, which can in turn lead to poor mental and physical health. This can create a vicious cycle where stigmatized behaviors lead to poor health outcomes, which then lead to further stigmatization. Overall, stigmatizing adverse health behavior is counterproductive and can have serious negative consequences. Instead, it is important to approach these issues with compassion and understanding, and to focus on providing effective care and support to those who need it. In this report, we have presented a relatively simple way to support these processes.

## Data availability statement

The raw data supporting the conclusions of this article will be made available by the authors, without undue reservation.

## Ethics statement

The studies involving humans were approved by the Ethics Committee of the Leibniz-Institut für Wissensmedien. The studies were conducted in accordance with the local legislation and institutional requirements. The participants provided their written informed consent to participate in this study.

## Author contributions

MB: Conceptualization, Data curation, Formal analysis, Methodology, Writing – original draft. ME: Conceptualization, Methodology, Writing – review & editing. JK: Conceptualization, Funding acquisition, Methodology, Project administration, Writing – review & editing.
