# Supplementary material for: Good reasons for bad behavior: a randomized controlled experiment on the impact of narrative reading and writing on empathic concern, perspective-taking, and attitude
Source: Front Public Health. 2024 Apr 5;12:1343225. doi: 10.3389/fpubh.2024.1343225 (PMC11026716; doi:10.3389/fpubh.2024.1343225)
Supplement: Supplementary file 1 [file Data_Sheet_1.docx]

Supplement 1:

Narrative text in the reading condition.

It was a rainy Monday morning. Joanie was on her way to work, thinking about everything she had to do today. She was pregnant, had two jobs and lived in the city. As she walked to the subway, the streets were already bustling. People were busy and minding their own business. She felt alone. Her husband was on a business trip, or so he said, and away for a few days. She could feel the baby kicking. Her back ached with every step she took. She was ready to stop being pregnant but felt like she wasn't ready to take care of a baby. She was stressed. The air was cold, and it was windy. She wore her favorite boots, which were comfortable and warm. She went to the usual café she went to every morning. It smelled delicious with fresh donuts and warm coffee and hot chocolate. She grabbed her coffee and set off again. As she passed a homeless man on the street, she thought about giving him a euro or two, but then she remembered that she didn’t really have a euro or two to spare because of the baby. It started to rain instead of just drizzle. Joanie thought about the homeless man and how he must deal with it all the time. “Now my hair will be wet, and my shoes will be wet all day at work,” she thought. She couldn't wait to sit down. Almost there. She finally made it to the steps and descended into the smelly, crowded, hot metro station. She got on and looked for a seat. She looked around and didn’t see a seat. She must have looked tired because a young man next to her got up and gave her his seat. Finally, she was on her way to work. It was only a 20-minute subway ride, then she could go inside and sit at her desk.
